# Multidrug resistant bacteria and associated risk factors of external ocular infections at University of Gondar tertiary hospital in Northwest Ethiopia

**DOI:** 10.1186/s12348-025-00541-2

**Published:** 2025-11-18

**Authors:** Mahliet Dereje, Abebe Birhanu, Meseret Mulu, Getachew Bitew, Alemnew Demissie, Aklilu Ambachew, Mulat Dagnew

**Affiliations:** 1 Department of Medical Microbiology, School of Biomedical and Laboratory Sciences, College of Medicine and Health Sciences, University of Gondar, P. O. Box 196, Gondar, Ethiopia; 2 Department of Ophthalmology, School of Medicine, College of Medicine and Health Sciences, University of Gondar, Gondar, Ethiopia

**Keywords:** External ocular infection, Bacterial profile, Multidrugresistance

## Abstract

**Background:**

Multidrug-resistant (MDR) bacteria are an escalating global public health concern and represent a cross-cutting issue affecting multiple sectors. In ophthalmic care, broad-spectrum antimicrobial agents are frequently prescribed empirically by healthcare professionals, often without culture-based evidence. This practice contributes to the development of drug-resistant pathogens. Therefore, routine surveillance of bacterial profiles and multidrug resistance in external ocular infections is crucial for effective treatment, prevention, and control efforts. The primary objective of this study was to determine the prevalence of multidrug resistance among bacterial isolates from external ocular infections and to identify associated risk factors.

**Method:**

A cross-sectional study was conducted among 360 external ocular infection suspected patients between May 1 and July 30, 2023, at the University of Gondar Comprehensive Specialized Hospital’s Tertiary Eye Care and Training Center. Systematic random sampling was employed to recruit participants. Sociodemographic and clinical data were collected using structured questionnaires. Ocular specimens were collected aseptically and processed using standard microbiological techniques according to CLSI. Data were entered into EpiData version 25 and analyzed using SPSS version 25. Bivariate and multivariate logistic regression analyses were performed to assess the risk factors, with a 95% confidence interval. A p-value of less than 0.05 was considered statistically significant. A total of 360 patients participated in the study.

**Results:**

Bacterial pathogens were isolated in 59.7% (215/360) of the external ocular infection samples. Gram-positive bacteria were the most frequently identified, comprising 46.7% (168/222) of isolates. *Staphylococcus aureus* was the most common isolate (43.7%, 97/222), followed by coagulase-negative *Staphylococcus* species (29.7%, 66/222), *Pseudomonas aeruginosa* (10.8%, 24/222), and *Escherichia coli* (5.4%, 12/222). The prevalence of methicillin-resistant *Staphylococcus aureus* (MRSA) was 21.6%. Overall, multidrug resistance was observed in 62.2% (138/222) of the isolates. Notably, dental infections were significantly associated with the presence of bacterial external ocular infections.

**Conclusions:**

This study highlights a high prevalence of bacterial and multidrug-resistant organisms in external ocular infections. *Gentamicin* and *ciprofloxacin* are effective antimicrobial agents against the isolated pathogens. These findings underscore the need for continuous monitoring of bacterial profiles and antimicrobial susceptibility patterns to support evidence-based antibiotic use and mitigate the rise of antimicrobial resistance in ocular infections.

## Introduction

The eye is the most vital sensory organ for vision. One of the common challenges affecting ocular health is eye infection, also known as ocular infection [1]. In a healthy eye, the density of the ocular surface microbiota is relatively low, and the diversity of microbial species is limited compared to other parts of the body [2]. This low microbial load is maintained through several mechanisms, including blinking, epithelial cell turnover, and the antimicrobial components of tears. Tears contain secretory immunoglobulin A (IgA), which prevents microbial adherence to the ocular surface and enhances neutrophil-mediated phagocytosis. Additionally, lysozyme exerts bactericidal activity, while lactoferrin inhibits microbial growth by sequestering essential nutrients [3]. Together, these defense mechanisms maintain a balance between microbial virulence and host immunity. However, infections can develop when the eye’s natural defenses are compromised due to factors such as systemic illness, immunosuppression, trauma, ocular surgery, contact lens wear, tear film deficiencies, and other external factors [4].

Although various ocular components may be exposed to pathogens, the external parts of the eye including the cornea, conjunctiva, and eyelids are most frequently affected [5, 6]. External ocular infections are commonly classified as conjunctivitis, keratitis, dacryocystitis, endophthalmitis, and blepharitis [7, 8]. Among these, conjunctivitis is the most prevalent. It can be categorized into three types: bacterial, allergic, and chemical. Bacterial conjunctivitis is contagious and often results from direct contact with contaminated eye makeup, facial creams, insects, or unclean hands. Allergic conjunctivitis occurs in susceptible individuals after exposure to allergens such as pollen, dust, fabrics, or animal dander, and may present seasonally or year-round. Chemical conjunctivitis results from exposure to irritants such as chlorine in swimming pools or environmental pollutants [9, 10]. Due to the overlap in clinical symptoms, distinguishing between bacterial and viral conjunctivitis based solely on signs is difficult, often leading to misdiagnosis and unnecessary antibiotic use [11]. Therefore, routine laboratory diagnosis and ongoing monitoring of ocular infections are critical to minimizing antimicrobial resistance. Although ocular infections can be caused by bacteria, viruses, or parasites, bacterial pathogens are responsible for approximately two-thirds of eye infections globally. Common bacterial agents include *Pseudomonas aeruginosa*, *Staphylococcus aureus*, *Streptococcus pneumoniae*, *Haemophilus influenzae*, and coagulase-negative *Staphylococcus* species (CoNS) [12]. Without timely and appropriate antibiotic treatment, infections may progress to serious complications such as corneal ulceration, perforation, scleritis, endophthalmitis, and even blindness [13].

The antibiotics most frequently used to manage bacterial ocular infections include β-lactams, aminoglycosides, fluoroquinolones, sulfonamides, and tetracyclines [14]. However, recent years have seen a significant rise in resistance to these agents. This trend is driven by factors such as inadequate surveillance, limited access to effective antimicrobials, misuse, and overuse of antibiotics. Multidrug-resistant (MDR) strains have become increasingly prevalent, particularly among *P. aeruginosa*, *Klebsiella pneumoniae*, *Escherichia coli*, *S. aureus*, and CoNS [7–9]. *P. aeruginosa*, in particular, has emerged as a highly resistant pathogen due to its ability to survive in distilled water, resist many disinfectants, and contaminate commonly used ophthalmic preparations such as artificial tears and ointments [15]. Evidence indicates a growing trend of resistance across multiple antibiotic classes commonly used in eye care [16]. Accurate treatment therefore depends on the proper identification of bacterial pathogens and determination of their antibiotic susceptibility profiles. This study aims to provide updated data on the bacterial profile of external ocular infections (EOIs), highlight current trends in multidrug resistance, and support improvements in patient care through targeted antimicrobial therapy.

## Materials and methods

### Study area

This study was conducted at the University of Gondar Comprehensive Specialized Hospital (UoGCSH) Tertiary Eye Care and Training Centre, located in Gondar City, Amhara National Regional State, Northwest Ethiopia. Gondar is approximately 750 km from Addis Ababa, the capital city of Ethiopia. Established in 2004, the UoGCSH Tertiary Eye Care Centre is the only tertiary-level eye care facility in Northwest Ethiopia, serving a catchment population of around 14 million people across 14 zones in the region [17]. The Centre provides a range of services including ophthalmic surgeries, inpatient care, and specialized outpatient services in Ophthalmology and Optometry. On average, the Optometry and Ophthalmology units attend to about 70 patients per day for services such as refraction assessments, minor and major eye surgeries, and various other diagnostic and treatment procedures.

Moreover, UoGCSH has a Clinical Laboratory that provides diagnostic services for patients. The laboratory comprises several units: Medical Microbiology, Hematology, Parasitology, Clinical Chemistry, Serology and HIV laboratory. The Medical Microbiology unit focuses on the culture of bacteria from various samples, including cerebrospinal fluid (CSF), stool, urine, blood, and other bodily fluids, such as eye discharge. It is equipped with a room for Gram staining and acid-fast staining. The laboratory maintains reference strains for quality control and conducts antimicrobial susceptibility testing. Additionally, it has a media preparation room, an autoclave room, and an inoculation room for sample handling, along with a culture reading room. A specialized TB culture room is available for culturing Mycobacterium tuberculosis and for molecular techniques, including GeneXpert, line probe assays, and other related assays.

### Study design and period

A hospital-based cross-sectional study was conducted over a three-month period, from May 1 to July 30, 2023.

### Source population

The source population included all patients attending the UoGCSH Tertiary Eye Care and Training Centre.

### Study population

The study population comprised patients presumptive of having external ocular infections who visited the Centre during the study period.

### Inclusion criteria

Patients presenting with signs of external ocular infections such as red eye, eye discharge, dry eye, or mucoid/mucopurulent secretions were included in the study.

### Exclusion criteria

Patients were excluded from the study if they had received antibiotic treatment within the two weeks prior to data collection, if they had experienced severe ocular trauma, or if they had undergone any recent ocular surgery at the time of recruitment.

### Variables

The study examined the prevalence of bacterial pathogens and their antimicrobial susceptibility patterns, which were treated as dependent variables. In contrast, sociodemographic characteristics such as age, residence, education level, and occupation served as independent variables. Additionally, various health-related factors were considered, including the presence of chronic illnesses, previous ocular or dental infections, a history of ocular trauma or surgery, the presence of dry eye disease, the use of artificial tears, a history of eye allergies, and self-medication practices.

### Sample size and sampling technique

The sample size were determined using single population proportion formula: n = Z^2^×P (1-p)/(d)^2^, where: n = the number of ophthalmic patients involved in this study; Z = Standard normal distribution value at 95% CI, which was 1.96; d = margin of error taken as 5%; P = the prevalence of bacterial ocular infections reported in Gondar Teaching Hospital, Ethiopia which were 62.4% (18). N = (1.96)^2^ × 0.624 (1-0.624.624.624)/(0.05)^2^= 360. Thus, a total of 360 patients were included in the study. A systematic random sampling technique was used to recruit participants. The estimated population during study period was 4200. To determine the sampling interval: 4200/360, which was rounded to 12. Therefore, every 12th patient was selected, with the first participant chosen using a lottery method.

### Data collection and processing

Data were collected using a structured questionnaire developed based on similar studies conducted [8, 9]. The questionnaire was initially prepared in English, then translated into the local language (Amharic), and subsequently back-translated into English to ensure consistency and accuracy. It included information on socio-demographic, behavioral, and clinical characteristics of the study participants.

Data collectors received two days of training on how to properly administer the questionnaire and collect specimens aseptically. Samples were collected by qualified healthcare professionals, including ophthalmologists and optometric nurses, under the supervision of the principal investigators. Gram staining and culture procedures were performed by the principal investigator at the Microbiology Laboratory of UoGCSH. All patients underwent external ocular examinations using a slit lamp biomicroscope to identify any signs of infection or inflammation. Following clinical assessment, socio-demographic data were collected using the structured questionnaire.

Standard operating procedures (SOPs), developed by the principal investigator, were followed throughout the study. Physical examinations and sample collections were performed by ophthalmologists or experienced optometric nurses. All clinical findings and demographic data were recorded using the structured data collection tool.

### Sample collection, handling and transportation of specimens

Samples were collected from the eyelid and conjunctiva using sterile rayon-tipped cotton swabs (Sunscreen, UK) moistened with sterile physiological saline. The swab was gently rolled over the inner lid margin, moving from the medial to lateral side and back again. For lid margins, sterile broth-moistened cotton swabs were used.

In cases of blepharitis, purulent material was collected from the lacrimal sac using dry, sterile swabs. This was done either by applying pressure over the lacrimal sac to allow reflux through the lacrimal punctum or by irrigating the lacrimal drainage system. For conjunctivitis, thick purulent discharge was collected, ensuring that neither the conjunctiva nor lid margins were touched. In dacryocystitis cases, pus was collected by pressing on the lacrimal sac or from surgically excised material.

Two swabs were collected from each participant, labeled, and inoculated into appropriate culture media. Swabs were transferred into a tube containing 2 mL of brain heart infusion broth (BHIB) and transported to the Microbiology Laboratory at UoGCSH within 30 min to minimize delays and ensure sample integrity.

### Bacterial isolation and identification

Samples were inoculated onto 5% sheep blood agar (BA) (Himedia, India), MacConkey agar (MCA) (Himedia, India), chocolate agar (CA) and mannitol salt agar (MSA) (Oxoid, Hampshire, UK). All media were incubated at 37 °C for 24–48 h. Swabs were also inoculated into sterile BHIB as an enrichment medium. MCA, MSA, and BA plates were incubated under aerobic conditions, while CA plates were incubated in a CO₂-enriched environment (5–10%). Plates were examined after 24 h, and those without visible growth were further incubated for up to 48 h. To enhance pathogen detection, samples were further processed using standard microbiological techniques, including Gram staining, colony morphology, and biochemical tests.

Gram-negative bacteria were identified using a series of biochemical tests: Kligler Iron Agar, citrate utilization, lysine decarboxylase, urease, motility, indole, oxidase, and X and V factor requirements (via satellite test).Gram-positive bacteria were identified based on hemolysis on blood agar, catalase and coagulase tests, bile solubility, and optochin sensitivity tests [18, 19].

### Antimicrobial susceptibility testing

Antimicrobial susceptibility testing was conducted for each bacterial isolate using the Kirby-Bauer disk diffusion method on Mueller-Hinton Agar (MHA) (Oxoid, Hampshire, UK). For fastidious bacterial pathogens, the antimicrobial sensitivity test was performed on MHA supplemented with 5% defibrinated sheep blood. This testing followed the guidelines set forth by the Clinical and Laboratory Standards Institute (CLSI), 2022 [20].

Briefly, 3–5 well-isolated colonies of the test organism were picked and suspended in sterile physiological saline. The suspension was gently mixed until it reached a turbidity equivalent to the 0.5 McFarland standards. The standardized suspension was then uniformly inoculated onto the surface of MHA plates using a sterile swab for non-fastidious organisms.

Antibiotic-impregnated disks were placed on the inoculated agar surface using a disk dispenser. The plates were incubated aerobically at 37 °C for 18–24 h. MRSA was screened using Cefoxitin disk diffusion method. Multidrug resistance (MDR) bacteria are bacteria that are resistant to two or more classes of antibiotics.

The following antibiotic disks, with their respective concentrations, were used for susceptibility testing: Ampicillin (AMP) 10 µg, Tetracycline (TE)−30 µg, Chloramphenicol (30 µg), Tobramycin (TOB), piperacillin (PIP) and imipenem (IMI). Ceftriaxone (CRO)−30 µg, Ciprofloxacin (CIP)−5 µg, Trimethoprim-sulphamethoxazole (SXT)−25 µg for Gram Negative bacteria while Ampicillin (AMP) 10 µg, Amoxicillin (AMC) −20 µg, Cefoxitin (FOX)−30 µg, Trimethoprim-sulphamethoxazole (SXT)−25 µg, Gentamicin (CN)−10 µg, Tetracycline (TE)−30 µg, Erythromycin (E) −15 µg, Ciprofloxacin (CIP) −5 µg, Ceftriaxone (CRO) −30 µg, Clindamycin (DA) −2ug, for Gram positive bacteria. Zone diameters were measured after incubation and susceptibility results were interpreted based on the CLSI, 2022 breakpoints [21].

### Data quality and laboratory quality control

The quality of the study was ensured throughout all phases: planning, data collection, laboratory analysis, and data entry. A two-day training session was provided to data collectors by the principal investigator, focusing on proper questionnaire administration and aseptic sample collection techniques. To evaluate the clarity, feasibility, and effectiveness of the questionnaire, a pilot study was conducted using 5% of the total sample size at the Felege Hiwot Referral Hospital Eye Unit prior to the main data collection.

The overall quality of laboratory procedures was maintained by strictly adhering to the Clinical and Laboratory Standards Institute (CLSI) guidelines and Standard Operating Procedures (SOPs) at every stage: pre-analytical, analytical, and post-analytical. The principal investigator closely monitored all aspects of data and specimen collection. Daily checks were conducted to ensure the completeness, accuracy, and clarity of the collected data.

The quality of ophthalmic specimens was ensured in accordance with SOPs. All culture media were prepared following the manufacturers’ instructions. Media sterility was verified by incubating 5% of prepared plates (Blood Agar, MacConkey Agar, Mannitol Salt Agar, and Mueller-Hinton Agar) at 37 °C for 24 h without specimen inoculation.

Reagent performance and media effectiveness were verified using known quality control strains: Catalase test: *Positive control: Staphylococcus aureus and Negative control: Streptococcus pyogenes.* For Gram staining reagents *S. aureus* (Gram positive) and *E. coli* (Gram negative) were used as quality control. For the bile solubility test positive control *S. pneumoniae*, negative control, *S. mitis* was used. Before use, all reagents and culture media were checked for expiry dates, and incubator and refrigerator temperatures were monitored daily to ensure proper storage and growth conditions.

The following American Type Culture Collection (ATCC**)** strains were used to assess the performance of culture media and antibiotic susceptibility testing throughout the study:*Staphylococcus aureus* (ATCC 6538, ATCC 25923), *Streptococcus pneumoniae* (ATCC 12977), *Streptococcus mitis* (ATCC 10712), *Escherichia coli* (ATCC 8739), *Streptococcus pyogenes* (ATCC 19615).

### Data processing and analysis

Data were cleaned, entered into EpiData version 4.7, and then exported to SPSS version 27.0 for statistical analysis. Bivariate and multivariate logistic regression analyses were performed to identify potential risk factors associated with bacterial external ocular infections (EOIs). P value less than or equal to 0.2 in bivariate analysis transferred into multivariate analysis and a p-value of < 0.05 was considered statistically significant.

### Patient and public involvement

Patients and the public were not involved in the design, implementation, or reporting of this study.

## Results

### Sociodemography of study participants

A total of 360 patients clinically diagnosed with external ocular infections were included in this study. The mean age of participants was 38.3 years. Among them, 55 participants (15.3%) were aged between 55 and 64 years. Of the total participants, 147 (40.8%) resided in rural areas, 179 (49.7%) were male, 209 (58.1%) were illiterate, and 197 (54.7%) were unmarried (Table 1.).

**Table 1.** Sociodemographic characteristics of patients with external ocular infection at UoGCSH, from May 1 to July 30, 2023

**Variables**	**Frequency**	**Percent**
Age (years)		
˂ 5	26	7.2
5-14	41	11.4
15-24	45	12.5
25-34	51	14.2
35-44	46	12.8
45-54	52	14.4
55-64	55	15.3
>65	44	12.2
Sex		
Male	179	49.7
Female	181	50.3
Residence		
Rural	147	40.8
Urban	213	59.2
Marital status		
No married	197	54.7
Married	163	45.3
Occupation		
Unemployed	4	1.1
Farmer	79	21.9
Civil servant	49	13.6
Private	51	14.2
Other	177	49.2
Educational level		
Illiterate	209	58.1
Primary	51	14.2
Secondary	45	12.4
College/university	55	15.3
Total	360	100

### Prevalence of external ocular infections

**Fig. 1:**
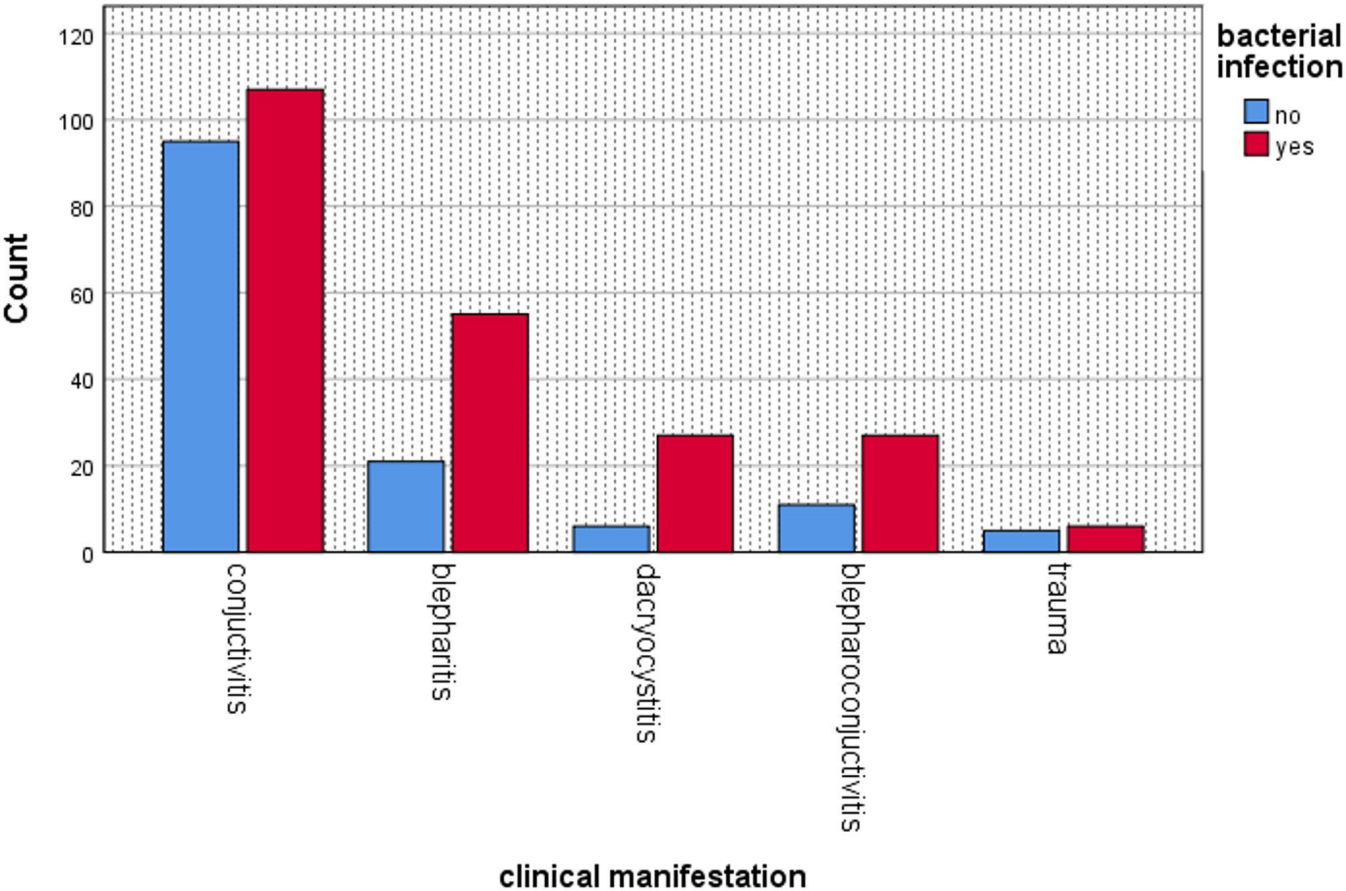
The magnitude of clinical Manifestation in external ocular infected patients at UoGCSH, from May 1 to July 30, 2023

Out of the 360 patients clinically diagnosed with external ocular infections, 215 (59.7%) were confirmed to have pathogenic bacterial isolates, with a 95% Confidence Interval (CI**)** of (56%–67%). The distribution of bacterial positivity by clinical manifestation was as follows: Dacryocystitis 26/33 cases (78.8%**)**, Blepharitis 56/76 (73.3%**)**, Blepharoconjunctivitis 27/38 (71.0%**)**, Trauma-related infections 6/11 (54.6%**)** and Conjunctivitis 107/201 cases (53.1%**)** (Fig. 1).

### Bacterial isolates and clinical features

A total of 222 bacterial isolates were recovered from the external ocular infection cases. Among these, 168 isolates (75.7%) were Gram-positive bacteria, while the remaining was Gram-negative. The predominant bacterial isolate was *Staphylococcus aureus*, accounting for 97 (43.7%) of all isolates, followed by Coagulase-negative *Staphylococcus* species (CoNS): 66 (29.7%), *Pseudomonas aeruginosa*: 24 (10.8%) and *Escherichia coli*: 12 (5.4%). Of the cases 7 patients had a double infection, each harboring more than one bacterial species. Staphylococcus aureus was the most commonly isolated organism in conjunctivitis cases (28.2%). In blepharitis, S. aureus and coagulase-negative staphylococci (CoNS) were predominant, accounting for 28.9% and 27.6% of cases, respectively. For dacryocystitis, S. aureus led with 33.3%, followed by Pseudomonas aeruginosa at 18.2%. These findings underscore the significant role of S. aureus in various external ocular infections (Table 2).

**Table 2:** Isolation rates of individual bacterial isolates from patients with external ocular infection at UoGCSH, from May 1 to July 30, 2023

Bacterial species	Conjunctivitis *n* = 202 N (%)	Blepharitis *n* = 76 N (%)	Dacryocystitis *n* = 33 N (%)	Blepharoconjunctivitis *n* = 38 N (%)	Trauma *n* = 11 N (%)	Total *N* (%)
*S. aureus*	57(28.2)	22(28.9)	11(33.3)	6(15.8)	1(9.1)	97
CoNS	26(12.9)	21(27.6)	3(9.1)	16(42.1)	0	66
*S. pneumoniae*	4(2)	0	0	0	1(9.1)	5
*P. aeruginosa*	9(4.5)	4(5.3)	6(18.2)	3(7.9)	2(18.2)	24
*E. coli*	6(3)	3(4)	2(6.1)	1(2.6)	0	12
*K. pneumoniae*	2(1)	4(5.3)	3(9.1)	0	2(18.2)	11
*Acinetobacter Spp.*	0	1(1.3)	1(3)	0	0	2
*Proteus spp.*,	3(1.5)	1(1.3)	0	1(2.6)	0	5
**Total**	107(53.1)	56(73.7)	26(78.8)	27(71)	6(54.6)	222

### Antibiotic resistance profile of Gram-positive bacterial isolates

A total of 168 Gram-positive bacterial isolates were tested for antimicrobial susceptibility. The highest susceptibility rates were observed for the following antibiotics: Chloramphenicol 91.1%, Clindamycin 89.9%, Gentamicin 81.0% and Trimethoprim-sulfamethoxazole (SXT) 76.8%. However, high resistance rates were noted for Tetracycline 75.0% and Ampicillin 73.8%.Among the Coagulase-negative *Staphylococcu****s*** species (CoNS**)** isolates, resistance to tetracycline and ampicillin was 75.8% and 69.7%, respectively.

Notably, the proportion of Methicillin-resistant *Staphylococcus aureus* (MRSA) among the *S. aureus* isolates was 21.6%. Detailed susceptibility patterns are presented in (Table 3).

**Table 3:** Antimicrobial susceptibility profile of Gram-positive isolates patients with external ocular infection at UoGCSH, from May 1 to July 30, 2023

Isolates	Susceptibility pattern	AMP	CXT	SXT	CAF	E	CN	TE	CIP	DA	CRO
S.aureus (97)	*R* I S	75(77.3) 2(2.0) 20(20.6)	21(21.6) 0 76(78.4)	19(19.6) 3(3.1) 75(77.3)	8(8.2) 0 89(91.8)	49(50.5) 0 48(49.5)	11(11.3) 0 86(88.7)	73(75.3) 5(5.2) 19(19.6)	12(12.4) 0 85(87.6)	10(10.3) 0 87(89.7)	16(16.5) 3(3.1) 78(80.4)
CoNS (66)	*R* I S	46(69.7) 2(3.03) 18(27.3)	37(56.1) 0 29(43.9)	14(21.2) 0 52(78.8)	7(10.6) 0 59(89.4)	45(68.2) 0 21(31.8)	16(24.2) 0 50(75.8)	50(75.8) 1(1.5) 15(22.7)	27(40.9) 0 39(59.1)	7(10.6) 0 59(89.4)	36(54.5) 0 30(45.5)
***S.pneumoniae*** **(5)**	**R** **I** **S**	**3(60)** **0** **2(40)**	**NA**	**3(60)** **0** **2(40)**	**0** **0** **5(100)**	**1(20)** **0** **4(80)**	**NA**	**3(60)** **0** **2(40)**	**NA**	**0** **0** **5(100)**	**NA**
**Total isolate** *N* = 168	**R** **I** **S**	**124(73.8)** **4(2.4)** **40(23.8)**	**58(34.5)** **0** **105(62.5)**	**36(21.4)** **3(1.8)** **129(76.8)**	**15(8.9)** **0** **153(91.1)**	**95(56.5)** **0** **73(43.5)**	**27(16.1)** **0** **136(81)**	**126(75)** **6(3.6)** **36(21.4)**	**39(23.2)** **0** **124(73.8)**	**17(10.1)** **0** **151(89.9)**	**52(31)** **3(1.8)** **108(64.3)**

*AMP* Ampicillin, *CTX* Cefoxitin, *SXT* Cotrimoxazole, *CAF *Chloramphenicol, *E* Erythromycin, *CN* Gentamycin, *TE* Tetracycline, *CIP* Ciprofloxacin, *CRO* Ceftriaxone, *DA* Clindamycin, *NA* Not applicable, *S* Susceptible, *I* Intermediate, *R* Resistance

###  Antimicrobial resistance profile of Gram-negative isolates

Most Gram-negative isolates were susceptible to imipenem 90.7%, and 66.7% were susceptible to ciprofloxacin, gentamicin, and trimethoprim-sulfamethoxazole, respectively. On the other hand, Gram-negative bacterial isolates showed resistance to ampicillin (86.7%) and tetracycline (60%). *Klebsiella pneumoniae* isolates showed a resistance rate of 63.6% to tetracycline. *Proteus spp.* also showed an 80% resistance rate to ampicillin (Table 4).

**Table 4:** Antimicrobial susceptibility test of Gram-negative isolates from patients with external ocular infection at UoGCSH, from May 1 to July 30, 2023

Organism isolated	ASP	CIP *n*(%)	SXT *n*(%)	AMP *n*(%)	TE *n*(%)	CN *n*(%)	CAZ *n*(%)	PIP *n*(%)	CRO *n*(%	TOR *n*(%)	IMI *n*(%)
*P. aeruginosa* (*N* = 24)	R I S	10(41.7%) 1(4.2%) 13(54.2%)	NA	NA	NA	11(45.8%) 0 13(54.2%)	11(45.8%) 0 13(54.2%)	17(70.8%) 0 7 (29.2%)	9(37.5%) 0 15(62.5%)	4(16.7%) 0 20(83.3%)	2(8.3%) 0 22(91.7%)
*E. coil* (*N* = 12)	R I S	3(25%) 0 9(75%)	4(33.3%) 0 8(66.7%)	10(83.3%) 0 10(83.3%)	9(75%) 0 3(25%)	4(33.3%) 0 8(66.7%)	NA	NA	4(33.3%) 0 8(66.7%)	NA	1(8.3%) 0 11(91.7%)
*K. pneumoniae* (*N* = 11)	R I S	3(27.3%) 0 8(72.7%)	4(36.4%) 0 7(63.6%)	11(100%) 0 0	7(63.7%) 0 4(36.4%)	3(27.3%) 0 8(72.7%)	NA	NA	4(36.4%) 0 7(63.6%)	NA	2(18.2%) 0 9(81.8%)
*Proteus spp.*, (*N* = 5)	R I S	1(20%) 0 4(80%)	2(40%) 0 3(60%)	4(80%) 0 1(20%)	1(20%) 0 4(80%)	0 0 5(100%)	NA	NA	1(20%) 0 4(80%)	NA	0 0 5(100%)
*Acinetobacter spp.*, (*N* = 2)	R I S	0 0 2(100%)	0 0 2(100%)	1(50%) 0 1(50%)	1(50%) 0 1(50%)	0 0 2(100%)	NA	NA	0 0 2(100%)	NA	0 0 2(100%)
Total (*N* = 54)	R S	18(33.3%) 36(66.7%)	10(33.3%) 20(66.7%)	26(86.7%) 4(21.1%)	18(60%) 12(40%)	18(33.3%) 36(66.7%)	11(45.8%) 13(54.2%)	17(70.8%) 7(29.2%)	18(33.3%) 36(66.7%)	4(16.7%) 20(83.3%)	5(9.3%) 49(90.7%)

*ASP* Antimicrobial susceptibility pattern, *CIP* Ciprofloxacin, *CN* Gentamycin, *n* number, *SXT* Cotrimoxazole, *TE* Tetracycline, *TOR* Tobramycin, *P* Piperacillin, *AMP* Ampicillin, *CAZ* Ceftazidime, *CRO* Ceftriaxone, *IMI* Imipenem, *NA* Not applicable *S* Susceptible, *I* Intermediate *R* Resistance

### Multidrug resistance profile of the bacterial isolates

Of the total bacterial species isolated, 138 (62.2%) were Multidrug resistant (MDR) with 95% CI [59–71]. Only 8 (3.6%) bacterial isolates were susceptible to all antibiotics tested. The MDR rates of CoNS, *S. aureus*, *P. aeruginosa* and *E. coli* were 46 (70%), 64 (66%),15(62.5%) and 7 (58.3%), respectively (Table 5).

**Table 5:** Antibiogram pattern of bacteria in patients with external ocular infection at UoGCSH, from May 1 to July 30, 2023

Antibiotics	S. aureus (*n* = 97)	CoNS (*n* = 66)	*S.pneumoniae* (*n* = 5)	*P. aeruginosa* (*n* = 24)	E. coil (*n* = 12)	*K.pneumoniae* (*n* = 11)	Proteus spp. (*n* = 5)	Total (*n* = 222)
DA + E + AMP CXT + AMP + TE DA + E + TE	13 6 7	2 5 1						
DA + E + AMP + TE SXT + E + AMP + TE SXT + E + CN + AMP	7 4 2	2 3 2	2					
CXT + SXT + E + CN + AMP	8	5						
CXT + CIP + SXT + E + CN + AMP CXT + CIP + DA + SXT + E+ CN + AMP CXT + CIP + CN + E + DA+ AMP + TE	9 7 1	11 9 6					1	
PIP + CAZ + TOR PIP + CAZ + TOR + CIP PIP + CAZ + TOR + CIP + IMI	0	0		11 2 2				
CAZ + CRO + IMI	0	0	0	0	7	3		
Total MDR	64(66%)	46(70%)	2(40%)	15(62.5%)	7(58.3%)	3(27.3%)	1(20%)	138(62.2%)

*AMP* Ampicillin, *CTX* Cefoxitin, *SXT* Cotrimoxazole, *CAF* Chloramphenicol, *E* Erythromycin, *CN* Gentamycin, *TOR* Tobramycin, *P* Piperacillin, *AMC* Amoxicillin Clavulanic acid, *CAZ* Ceftazidime, *CRO* Ceftriaxone, *IMI* Imipenem, *MDR* non-susceptible to $\:\ge\:$3 antimicrobial classes

### Bivariate and multivariate analysis of factors associated with external ocular infections

In multivariate analysis, external ocular infection was significantly associated with the presence of dental infection (AOR = 2.53, CI = 1.530–4.184) and eye allergy (AOR = 3.474, CI = 2.086–5.786). Participants who had a dental infection were 2.5 times more likely to have a bacterial external ocular infection than participants who did not have a dental infection. Likewise, participants with a previous eye allergy were 3.5 times more likely to develop a bacterial external ocular infection than participants without an eye allergy (Table 6).

**Table 6:** Bivariable and multivariable analysis of factors associated with bacterial external ocular infection in patients at UoGCSH, from May 1 to July 30, 2023

**Risk factors**	External ocular infection		COR (95% CI)	*P*-Value	AOR (95% CI)	*P*-Value
	**Yes**	**No**				
Sex						
Male	113(63.1%)	66(36.9%)	0.884(0.578–1.353.578.353)	0.571	NA	NA
Female	109(60.2%)	72(39.8%)				
Residence						
Urban	124(58.2%)	89(41.8%)	1.435(0.926–2.224.926.224)	0.106*	1.435(0.926–2.224.926.224)	0.106
Rural	98(66.7%)	49(33.3%)				
Presence of dry eye						
Yes	63 (54.8%)	52(45.2%)	1.526(0.972–2.396.972.396)	0.066*	0.481(0.281–0.822.281.822)	0.066
No	159(64.9%)	86(35.1%)				
Artificial tear user						
Yes	37(52.1%)	34(47.9%)	1.635(0.968–2.760.968.760)	0.066*	1.111(0.413–2.993.413.993)	0.835
No	185(64.0%)	104(36.0%)				
Presence of dental infection						
Yes	124(77.0%)	37(23.0%)	0.290(0.183–0.459.183.459)	0.001*	2.530(1.530–4.184.530.184)	0.001*
No	98(49.2%)	101(50.8%)				
Previous ocular disease						
Yes	106(62%)	65(38%)	1.026(0.671–1.571.671.571)	0.905	NA	NA
No	116(61.4%)	73(38.6%)				
Previous eye surgery						
Yes	17(63%)	10(37%)	0.942(0.418–2.121.418.121)	0.885	NA	NA
No	205(61.6%)	128(38.4%)				
History of self-medication						
Yes	90(60.8%)	58(39.2%)	1.063(0.691–1.637.691.637)	0.780	NA	NA
No	132(62.3%)	80(37.7%)				
Previous ocular trauma						
Yes	35(70%)	15(30%)	0.652(0.341–1.243.341.243)	0.194*	1.784(0.862–3.694.862.694)	0.119
No	187(60.3%)	123(39.7%)				
Presence of eye allergy						
Yes	151(75.5%)	49(24.5%)	0.259(0.165–0.405.165.405)	0.001*	3.474(2.086–5.786.086.786)	0.001*
No	71(44.4%)	89(55.6%)				
Contact lens						
Yes	11(52.4%)	10(47.6%)	0.667(0.276–1.615.276.615)	0.370	NA	NA
No	211(62.2%)	128(37.8%)				
Completely use of drug						
Yes	126(60.3%)	83(39.7%)	0.870(0.565–1.340.565.340)	0.527	NA	NA
No	96(63.6%)	55(36.4%)				
Use of soap						
Yes	94(59.9%)	63(40.1%)	1.144(0.746–1.755.746.755)	0.538	NA	NA
No	128(63.1%)	75(36.9%)				
Traditional medication						
Yes	18(66.7%)	9(33.3%)	0.791(0.551–2.900.551.900)	1.265	NA	NA
No	204(61.3%)	129(38.7%)				

Statistically significant association (P-value < 0.05)

*COR* Crude Odds Ratio, *AOR* Adjusted Odds Ratio, *CI* Confidence interval, *NA* Not applicable

## Discussion

External ocular infection is a major public health problem in Ethiopia and globally (7,8,9, 16). In this study, the overall prevalence of bacterial external ocular infections was 59.7% (215/360), which aligns with findings from Addis Ababa 59.4% [9], and previous reports in Gondar 62.4% and 58.3% [17, 21]. However, this finding showed higher prevalence than the study conducted in Hawassa (48.8%) [8], and Italy 32% [22] but lower than those documented in Jimma 74.7% [23] and Ghana 95.1% [24]. These discrepancies may be due to variations in study design, geographical region, socioeconomic conditions, public awareness, and infection prevention practices.

In our study, conjunctivitis was the most frequent type of EOI, followed by blepharitis, blepharoconjunctivitis, dacryocystitis, and trauma-related infections. This trend is consistent with studies from Hawassa [8], Gondar (22), Ghana [24], and Bahir Dar [25]. However, other studies conducted in Gondar and Borumeda found blepharitis to be the most common EOI [26, 27]. The difference might be due to regional differences in disease patterns.

Gram-positive cocci were the dominant bacterial isolates, accounting for 75.7% (168/222) of infections. This finding is supported by studies from Gondar [21], Jimma (23), Ghana [225], Bahir Dar [25], Yemen [16], and Italy (81.1%) [3]. *Staphylococcus aureus* was the most frequently isolated organism, particularly in cases of conjunctivitis, blepharitis, and blepharoconjunctivitis, corroborating results from Addis Ababa [9], Jimma [23], Ghana [24], and Bahir Dar [25]. In contrast, *Coagulase-negative Staphylococcus* species (CoNS) were most commonly associated with dacryocystitis in other studies from Gondar [26], Italy [22], and Borumeda [27] possibly due to differences in sample collection sites and laboratory methods. In many studies, the predominant bacteria are Gram-positive cocci. This is might be due to contamination of the eye caused by transmission of bacteria from normal skin flora by touching eyes with hands, cataract extraction, lens implantation, and use of contact lens (9).

Gram-negative bacteria comprised 24.3% of isolates, which is comparable to findings in Hawassa (38.5%) [8], Ghana [24], and Bahir Dar (33.3%) [25]. *Pseudomonas aeruginosa* was the predominant Gram-negative isolate in this study, consistent with findings from Yemen [16], Gondar [26], Jimma [22], Ghana [24], and Italy [3]. However, other studies in Hawassa [8], Gondar [21], and Bahir Dar [25] identified *Klebsiella pneumoniae* as the most common Gram-negative pathogen. The difference might be due to sample difference, time, geography and different laboratory techniques.

Bacterial resistance is a growing challenge in Ethiopia, as observed in this study. High resistance rates were noted for ampicillin (75.8%), piperacillin (70.8%), and tetracycline (72.7%), comparable to findings in Jimma [22], and higher than reports from Hawassa (52.5%) (8) and Bahir Dar (42.1%) [25]. This is due to antimicrobial resistance is increasing from time to time due to misuse of antibiotics in Ethiopia.

Gram-positive bacteria exhibited particularly high resistance to tetracycline (75%), ampicillin **(**73.8%), and erythromycin (56.5%), consistent with reports from Jimma [23]. Meanwhile, these isolate showed high susceptibility to clindamycin (89.9%), gentamicin (81%), trimethoprim-sulfamethoxazole (76.8%), and ciprofloxacin (73.8%), aligning with similar trends in Hawassa [8] and Bahir Dar [25].

*S. aureus* showed resistance to ampicillin (77.3%) and tetracycline (75.3%), possibly due to β**-**lactamase production and altered penicillin-binding proteins [21]. The prevalence of MRSA was 21.6%, consistent with a previous study in Gondar (24%) [19], but higher than rates reported in Hawassa (6.7%) and Bahir Dar (16.9%) [25]. The presence of MRSA was determined by resistance to cefoxitin, though more accurate detection could involve identification of the mecA gene or penicillin-binding protein 2a (PBP2a) [28, 29]. This rising MRSA prevalence reflects the growing challenge of antimicrobial resistance in low-resource settings.

CoNS isolates also demonstrated high resistance to tetracycline (75.8%), penicillin (72.7%), and ampicillin (69.7%), which is consistent with earlier findings from Gondar and Bahir Dar [21, 25, 26]. If not effectively treated, EOIs can result in serious complications including vision loss (23). *Streptococcus pneumoniae* isolates showed high resistance to ampicillin, tetracycline, and trimethoprim**-**sulfamethoxazole, similar to studies in Hawassa (65%) [8] and Bahir Dar (66.6%) [25].

Gram-negative isolates showed the highest resistance to ampicillin (86.7%) and tetracycline (60%), supported by reports from Addis Ababa [9] and Gondar [21]. *P. aeruginosa* was notably resistant to piperacillin (70.8%), yet highly susceptible to imipenem **(**91.7%) and tobramycin (83.3%), in line with previous findings in Gondar [17, 26].

All *K. pneumoniae* isolates were resistant to ampicillin (100%), and showed resistance to tetracycline **(**63.6%) and **i**mipenem (19.2%), suggesting increasing resistance even to last-resort drugs. *E. coli* isolates were resistant to tetracycline (75%) and ampicillin (83.3%), consistent with previous studies in Borumeda [27]. *Proteus* spp. were resistant to ampicillin (80%) but susceptible to gentamicin (100%), and tetracycline and ciprofloxacin (80%), as reported in Hawassa and Addis Ababa [8, 9].Widespread overuse and misuse of antibiotics in Ethiopia likely contributes to the emergence and spread of resistant strains.

The overall MDR rate in this study was 62.2% (138/222), comparable to the 66.4% reported in Gondar [26], but higher than the 45.2% found in Bahir Dar [23], and lower than the 87.1% reported in another Gondar study [30]. Differences may result from varying antibiotic testing panels and methodologies.

Among Gram-positive bacteria, CoNS exhibited the highest MDR rate at 70%, which is higher than previous reports from Gondar (44.4%, 59.4%) [21, 26] and Bahir Dar (45.5%) [25]. Among Gram-negative bacteria, *E. coli* had an MDR rate of 58.3%, similar to Gondar (50%) [21], but higher than Bahir Dar (20%) (26). *K. pneumoniae* showed an MDR rate of 27.3%, similar to Hawassa (33.3%) [8], but lower than other studies from Gondar (62.5%, 77.7%) [21, 26] and Bahir Dar (64.3%) [25]. These differences may reflect variations in testing standards, time of study, and treatment practices.

This study identified eye allergy as a significant predictor of EOIs, consistent with findings from Bahir Dar [25]. Allergic inflammation may compromise the ocular surface barrier, increasing vulnerability to infection. Frequent eye rubbing in allergic patients may also introduce pathogens by disrupting the normal microbial flora [31]. Dental infections were also associated with EOIs. This is supported by studies from India [32] and Istanbul [33], suggesting that oral bacteria can spread to the eyes via contaminated hands or hematogenous routes, and leading to secondary ocular infections.

## Conclusion and recommendations

Conjunctivitis is the most common form of external ocular infections. *S. aureus* was the overall predominant isolated pathogen, followed by CoNS, *P. aeruginosa* and *E. coli.* Multidrug resistant bacteria are high in external ocular infection.

Early detection and appropriate treatment are important to minimize permanent vision loss. Continuous monitoring of the bacterial profile and their drug resistance patterns is crucial for the prudent use of antibiotics and reducing the emergence of drug resistance. Therefore, prudent use of antibiotics in external ocular infections is essential for ocular bacterial infections. There is a need for critical interventions like educating patients about proper hand hygiene, avoiding eye contact, proper use of antibiotics, and the importance of notifying healthcare providers if they experience eye-related symptoms or concerns.

### Strengths and limitations of the study

This study has done Carbapenems resistance, Methicillin resistance and multidrug resistant bacteria which were not done in the previous research. Due to lack of reagents and culture media, this study did not isolate *Chlamydia trachomatis* which is the common cause of trachoma in developing countries.

## Data Availability

All data generated or analyzed during this study are included in this published article.

## Abbreviations

ASP: Antimicrobial Susceptibility Pattern
AST: Antimicrobial Susceptibility Test
ATCC: American Type Culture Collection
AMR: Antimicrobial resistance
BA: Blood Agar
CA: Chocolate Agar
CLSI: Clinical Laboratory Standard Institution
CoNS: Coagulase-negative Staphylococcus spps
EOIs: External ocular infections
IgA: Immunoglobulin A
MCA: MacConkey Agar
MDR: Multi Drug Resistance
MHA: Muller Hinton Agar
MRSA: Methicillin resistance S. aureus
MSA: Mannitol Salt Agar
UoG: University of Gondar
UoGCSH: University of Gondar Compressive specialized hospital
USA: United States of America
WHO: World Health Organization
